# Sleep supports the consolidation of newly learned statistical concepts

**DOI:** 10.1177/17470218251317885

**Published:** 2025-01-23

**Authors:** John J Shaw, Marie-Josee Bisson

**Affiliations:** 1 Edge Hill University, Ormskirk, UK; 2 De Montfort University, Leicester, UK

**Keywords:** Sleep, numerical cognition, memory, comparative judgement

## Abstract

Within mathematical cognition, the development of conceptual knowledge is seen as critical to developing understanding. Sleep has been well established to play a role in the consolidation of newly learned information and schema-based information but has yet to be explored within mathematical cognition. Across three experiments, participants (*N* = 167) were assigned to a sleep or wake group and then viewed lectures on either *p*-values, *t*-test, or *z*-scores. The sleep group watched the lecture at 9 p.m., completed an immediate recall task to explain the concept, then a second recall task 12 hr later at 9 a.m. The wake groups watched the lecture at 9 a.m. and completed an immediate recall task then a second recall task 12 hr later at 9 p.m. Written responses were then assessed using a comparative judgement task by subject experts. Across all three experiments, results showed that participants in the sleep group retained their knowledge from the immediate recall to 12 hr later, whereas in the wake group, participants’ knowledge declined significantly between sessions. These results suggest that sleep may be involved in an important process of maintaining the information learned from statistical concepts.

An ever-growing body of research has demonstrated that post-encoding sleep has an important role in consolidation of newly learned information for both declarative memory such as verbal (Clemens et al., 2005; Lahl et al., 2008) and linguistic (Dumay & Gaskell, 2007; Tamminen et al., 2013), as well as procedural memory such as motor (Backhaus & Junghanns, 2006; Walker et al., 2003) and spatial (Ferrara et al., 2008; Wamsley et al., 2010) memory. Sleep is considered to offer a stabilising effect of memory compared with an equivalent wake interval, offering protection against external interference (Ellenbogen et al., 2006; Batterink & Paller, 2017), as well as enhancing recall performance (Gais et al., 2002; Plihal & Born, 1997). There are two general theories for this consolidation effect; interference hypotheses suggest that sleep plays a passive role in memory consolidation, offering a period where hippocampal resources are available for consolidation instead of learning, meaning that external stimuli cannot retroactively interfere with newly encoded information (Ellenbogen et al., 2006; Mednick et al., 2011), but beyond this, sleep offers no active consolidation. The second theory proposes a more active role of sleep, such as the broadly accepted active systems consolidation theory, drawing on trace transformation theory whereby information is initially encoded as both a high-fidelity hippocampal-based episodic representation serving as a fast-learning temporary store, and a weaker encoded neocortical representation as the slow-learning long-term store (Winocur & Moscovitch, 2011). As the cortex takes longer to form connections between memory elements, the hippocampus acts as a binding point for neocortical representations, with repeated reactivation of the hippocampal memory representation in the same order as during learning (Ji & Wilson, 2007; Skaggs & McNaughton, 1996). Under active systems consolidation theory, it is during sleep, and in particular non-rapid eye movement (NREM) sleep, that this repeated hippocampal reactivation occurs (Diekelmann & Born, 2010; Rasch & Born, 2013), leading to a qualitative reorganisation of the representation (Born & Wilhelm, 2012; Cairney et al., 2018), shifting the representation from a primarily hippocampal-based episodic representation to the neocortical-based semantic representation. To summarise, sleep’s role both strengthens storage of episodic memories in the short term (~ 12 hr), as well as transforming them to a gist-based representation over the longer term.

The learning of new conceptual material and the impact of sleep on this has been previously explored; Hennies et al. (2016) identified sleep spindle density as a predictor of semantic knowledge consolidation, and more recently Graveline and Wamsley (2017) observed that sleep can aid novel concept learning. Within their study, participants in a sleep group or a wake group were trained on classifying dot patterns into specific categories and 12 hr later tested using a recognition task on previously seen patterns and novel patterns that fit the concept categories developed. Participants in the wake group experienced a significant decline in performance over the delay, and though those in the sleep group did not experience an improvement, they maintained performance at a similar level to Session 1, demonstrating that sleep does support consolidation of newly learned concepts within a short time period.

However, the benefit of sleep is not universal, as type of stimuli also plays a key role. Previous literature suggests that sleep preferentially benefits weakly encoded declarative memories (e.g., static stimuli such as paired associates, wordlists, or images such as those used in Graveline & Wamsley, 2017; Hennies et al., 2016) that often use cued-recall or recognition tasks, rather than strongly encoded stimuli, such as stories (Schoch et al., 2017) that often rely upon free recall. When compared to naturalistic free-recall episodic tasks that use stories, the evidence of sleep’s benefit is much more mixed. Wagner et al. (2001) used naturalistic stories in a sleep study with either an emotional topic (e.g., paraplegia), or a neutral topic (e.g., bronze making). Each story was recalled immediately after exposure and once after a 3 hr delay containing sleep or wakefulness. Those who slept retained more content from the emotional stories scored via the number of content words recalled, though this did not replicate for the neutral stories, with Wagner et al. suggesting sleep benefits depend upon the emotionality of the content. In contrast, Aly and Moscovitch (2010) investigated the impact of age and sleep on neutral stories and observed a clear sleep benefit. Younger and older adults completed an episodic memory task, the Logical Memory section of Wechsler Memory Scale III, whereby participants were scored on recall of paragraph-length stories. They observed that episodic memory recall was superior in the sleep group, suggesting a beneficial role of sleep in the consolidation of episodic memory. However, more recently, van Rijn et al. (2021) found no significant effect of sleep on free-recall memory, a result replicated by Cohn-Sheehy et al. (2022) who found no effect of sleep in free story recall of neutral stories. In contrast, Coutanche et al. (2020) found a beneficial effect of sleep in free recall of details from an episodic visual scene (wildlife videos) compared with an equivalent period of wake, further emphasising that it may be that specific methodological differences such as stimuli format or repeated testing (Cohn-Sheehy et al., 2022; van Rijn et al., 2021; See Berres & Erdfelder, 2021) may account for these differences. Furthermore, these experiments have often used an all-or-nothing approach to scoring of free-recall answers, whereby points were scored for correctly recalling content, whereas inferences or errors were discarded, losing some nuance of participants’ answers. Furthermore, these studies often utilise topics that are not typical in the education system, as such their presentations often are unlike what one would normally experience (e.g., lectures, podcasts, or instructional videos).

One element that has yet to be investigated is the role of sleep in the learning of mathematical concepts. Within the field of mathematical cognition, there are broadly two types of mathematical knowledge: procedural knowledge and conceptual knowledge. Although in wider psychology, procedural knowledge refers generally to knowledge of physical actions (e.g., riding a bike or observed using the finger-tapping task), within mathematical cognition, procedural knowledge refers more specifically to the understanding of how to apply a sequence of operations successfully, or “knowing how-to.” Likewise, in wider psychology, concepts can be defined as a set of related stimuli that allows the abstraction of generalised knowledge of a category (Medin & Smith, 1984), and in mathematical cognition, conceptual knowledge refers to the understanding of abstract concepts, relationships, and principles between information, or “knowing why” (Hiebert & Lefevre, 1986). Although the precise definition of conceptual knowledge is somewhat open to interpretation and change over time (Crooks & Alibali, 2014), for the purpose of this article, we use the definition of conceptual understanding by Rittle-Johnson and Schneider (2014) as “knowledge of abstract concepts.” For example, for fractions, conceptual knowledge is knowing what fractions are and what 3/5 represents, whereas procedural knowledge may be knowing how to convert that from 6/10 or converting 3/5 to a percentage. The iterative developmental model by Rittle-Johnson and colleagues suggests that advances in one knowledge domain lead to advances in the other (Rittle-Johnson et al., 2001; Rittle-Johnson & Siegler, 1999). However, within educational policy, there is a unidirectional conceptual to procedural knowledge perspective, requiring conceptual knowledge built over an extended period ahead of any procedural practice (Baroody, 2003), with this approach forming a key part of the recent National Council of Teachers of Mathematics (NCTM, 2014) report. Although this unidirectional approach is debated within academia (Rittle-Johnson et al., 2015), it is well established that building strong conceptual knowledge is seen as highly beneficial to procedural knowledge and developmentally important.

Yet, despite the importance placed on numeracy skills, there is concern regarding numeracy levels. A recent survey conducted by the Organisation for Economic Co-Operation and Development (OECD, 2013) found that across 24 countries ~ 20% of adults had low numeracy levels to the extent of struggling with concepts such as percentages and decimals. In the United Kingdom, it is estimated that ~ 24% of adults have numeracy levels below that which is needed for everyday functioning, struggling to understand food prices/discounts or household bills (Department for Business, Innovation and Skills, 2012). Within the United States, 35% of senior students leaving school were below the expected proficiency in mathematics (Kena et al., 2016). This is particularly problematic as multiple studies have linked low-level numerical skills to decreased wealth, health, and quality of life (C. Hudson et al., 2009; OECD, 2013; Parsons & Bynner, 2013; Reyna et al., 2009). As such, the need to develop an understanding of mathematics, both the concepts underlying it and the ability to apply it, is crucial to positive health outcomes, with efforts being made to address this (B. Hudson et al., 2015; Wittmann, 2021). An area of mathematics that receives strong focus in adults is that of statistics. Statistics, and by extension statistics classes, are often reported to be anxiety-provoking (Williams, 2013) and the concepts taught being difficult to learn (McGrath, 2014), with a review article by Garfield and Ben-Zvi (2007) highlighting key statistical concepts such as centre, variability, and distributions being particularly difficult for students. A qualitative study by McGrath (2014) reported that while some participants struggled with the mathematics behind statistics, many participants reported difficulty with understanding the core concepts of statistics and where they are applicable. This in turn provokes further statistics anxiety, impeding learning and academic performance, creating a negative feedback loop around statistics.

The focus of this article is to investigate the role of sleep in consolidating newly learnt statistics-based concepts with a particular focus on free recall to prevent any cueing of knowledge from a multiple-choice test. Within this article across three experiments, participants view a lecture introducing a new statistical concept (*p*-values, *z*-scores, and *t*-test), with these topics chosen due to no prior exposure to the concepts. Within Session 1, participants were presented with a lecture on the specific topic and then completed an immediate free-recall task measuring their conceptual knowledge. Twelve hours later after a delay that either contained sleep or wake, participants completed a second recall task.

To address the limitations of previous experiments that have used an all-or-nothing scoring measure for free-recall answers (Aly & Moscovitch, 2010; van Rijn et al., 2021; Wagner et al., 2004), these submitted answers are then subject to a comparative judgement (CJ) task as a measure of conceptual understanding (Bisson et al., 2016, 2020; I. Jones et al., 2019). CJ is based on the principle that humans are better at comparing two items against one another than against a set criterion (Thurstone, 1994). Under this method, markers who are experts in the topic are presented with a pair of participant answers and must decide which one is better. This is then repeated until markers are presented with all answers. When engaging with a CJ task, markers are not asked to look for anything particular in the answers. Rather, they rely on their collective knowledge of what makes “*a good answer*,” grounding the approach in the collective judgement of a community of experts rather than predetermined definitions and rubrics. CJ has been used across disciplines, including cross-linguistic similarity (Bisson, 2023), visual complexity (S. R. Jones et al., 2020), and areas of deprivation (Seymour et al., 2022), with a recent experiment by Bisson et al. (2016) comparing students’ understanding of three topics in mathematics (*p-*values, derivatives in calculus, and letters in algebra) as measured using CJ from a group of subject experts to established psychometric scales (e.g., RPASS-7, see Lane-Getaz, 2013). Across all topics, they observed a high level of validity when comparing CJ to validated scales and other performance indicators such as course grades, along with high reliability of the judgements made, suggesting that CJ is a valid alternative for measuring conceptual knowledge. Overall, CJ is an efficient, reliable, and precise measurement method (see I. Jones & Davies, 2024) and is particularly suited to measuring difficult to operationalised constructs such as conceptual understanding where a bespoke instrument does not exist or has not been validated for a particular context or population, thus making it suitable for measuring learning of a concept. With CJ, there is no need to develop many items such as in multiple-choice tests and refine and validate a scale. One open-ended question about the concept of interest is needed, and participants’ answers are then presented in pairs to judges with the judge picking the better answer of the two. This is repeated across all answers, with answers being presented multiple times.

Once all answers have been judged several times across markers, the Bradley–Terry model, a probabilistic model based on maximum-likelihood estimation used across disciplines such as education (Roose et al., 2019), psycholinguistics (Bisson, 2023), and mathematics (Davies et al., 2021), is used to calculate a unique and precise score for each participant’s answer, which can then be used in further statistical analyses (Bradley & El-Helbawy, 1976; Bradley & Terry, 1952; Firth, 2005; I. Jones & Davies, 2024; Turner & Firth, 2012). These parameter estimates indicate the probability (*P*) of one script (*A*) being rated as better than another (*B*) based on their knowledge (otherwise referred to in literature as the ability or skill parameter; *e*) as expressed in Equation 1, with a positive score indicating a higher probability of being selected as the better answer when compared to any other script, whereas a negative score indicates a lower probability of being selected as the better answer. This parameter estimate can then be subjected to psychometric checks, hypothesis testing, and other procedures similar to test scores generated via traditional methods:

(1) $$P(A\;beats\;B)\;=\;\frac{e^{\lambda \_A}}{e^{\lambda \_A}\;+\;e^{\lambda \_B}}$$

Importantly, compared to using raw scores from the CJ (number of wins and losses for each answer script) that rewards the winner and punishes the loser equally for each comparison, the Bradley–Terry model weights the quality of each answer by considering small differences between pairs of scripts. For example, when there is a comparison between two “good” scripts as measured by the previously calculated probability of being chosen, the Bradley–Terry model takes into account the small difference between the scripts to “reward” the winner but “punish” the loser only slightly. Reliability of the measurement scale is assessed through the Scale Separation Reliability (SSR), analogous to Cronbach’s α, but measuring the degree scores are separated, indicating clear differences between answers, with an SSR of ⩾ 0.7 considered acceptable (Bramley, 2007; I. Jones & Davies, 2024). Calculating unique parameter estimates for each answer means that answers are not just ranked but form a scale where relative distances between items are intervals (I. Jones & Davies, 2024).

For each experiment, we hypothesised that there would be a significant interaction between session and group, with no significant difference in conceptual understanding between the sleep and wake group in Session 1 (immediate recall), but a significant difference based on the group in Session 2 (12 hr recall). It was predicted that the sleep group in Session 2 would display higher levels of conceptual knowledge compared with the wake group. The same procedure was used in all three experiments and the data was only analysed once the three experiments had been conducted. We, therefore, present the procedure and results for each experiment before discussing the results of all three experiments together.

## Experiment 1: understanding of *p-*values

### Participants

A total of 72 participants (41 females, *M* = 23.1, *SD* = 6.2) were recruited either through prolific.co (35 participants) or from a first-year undergraduate Psychology statistics module and were unfamiliar with the concept ahead of the experiment. None of the participants from prolific.co had completed a degree in Psychology. All participants were UK-based and had English as their first language. Participants recruited via prolific.co were paid £8.10 upon completion of the second session, whereas undergraduate students received course credits. Undergraduate participants were told that participation in the study was entirely voluntary and did not affect their undergraduate statistics module. The experiment was approved by the University Ethics Board, and written consent was obtained for all participants. Participants did not complete any other experiment within this article. An a priori power analysis conducted using G*Power (3.1.9.7) for a mixed-effects interaction determined that 54 participants would detect a medium effect size (f = 0.25) with the alpha set to .05 at a power level of 0.95. We recruited a slightly higher number of participants to account for participant dropping out or failing some of the data quality checks. All participants were instructed not to have caffeine and/or alcohol during the period of the experiment, or 12 hr prior, and to refrain from naps during the period of the experiment.

### Stimuli

Participants were presented with four questionnaires ahead of the lecture focusing on their sleep and their math anxiety. These were:

#### Demographic questionnaire

Participants completed a demographic questionnaire asking their age, gender, if they have been diagnosed with a neurodivergent condition, and the highest level of education accomplished.

#### Pittsburgh Sleep Quality Index

We checked that the participants’ sleep was adequate using the Pittsburgh Sleep Quality Index (PSQI; Buysse et al., 1989). This scale uses the total score of seven components with each item on the scale scored 0 (difficult) to 3 (severe difficult), and is widely recognised as a reliable scale, with Cronbach’s α of .83 and a test–retest correlation of .85. Although in the original article, a score of 5 was used to identify poor sleepers, within the current experiment participants who scored 7 or above were removed from the analysis, a cut-off that has identified poor sleepers in clinical practice (Beck et al., 2004; Carpenter & Andrykowski, 1998; Fichtenberg et al., 2001). Analysis was initially conducted with participants with a PSQI score ⩽ 5, but results did not differ from a cut-off of ⩽ 7, so this formed the final dataset.

#### Mathematics Self-Efficacy and Anxiety Questionnaire

This validated and reliable measure assessed self-efficacy and anxiety concerned with mathematics (May, 2009). Items were originally written to be directed towards self-efficacy and anxiety in the mathematics of a calculus class. All items were adjusted to focus on general mathematics ability. The 16 items were on a 5-point Likert-type scale (1 “*Never*” to 5 “*Usually*”), such as “I believe I am the kind of person who is good at mathematics.” Items on each subscale were summed for total scores where higher scores represented greater mathematics self-efficacy and lower anxiety.

#### Stanford Sleepiness Scale

Participants also completed the Stanford Sleepiness Scale (SSS; Hoddes et al., 1973) to measure current sleepiness levels at the time of the session. SSS is a single-item measure requiring respondents to select one of seven statements best representing their levels of perceived sleepiness, with a higher number representing a greater level of sleepiness.

#### Lecture on *p*-values

The recorded lecture on *p*-values was taken from the first-year undergraduate Psychological Research Methods module at the researchers’ University. The module is designed for students with no statistical background to become familiar with common statistics in psychological research. The lecture, presented in English, was 17 min 29s long and addressed what *p*-values are, how they are used in psychology, and how to interpret them. Before and after the lecture, participants were asked a single, open-ended question:

Explain what a *p*-value is and how it is used to someone who hasn’t encountered it before in as much detail as you can. If you don’t know what a *p*-value is, please write “I don’t know.”

If participants indicated prior knowledge of the topic before the lecture, they were excluded from the analysis.

### Procedure

**Figure 1.**
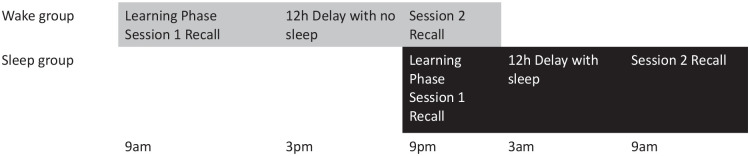
Experimental design for Experiment 1.

The study consisted of a learning phase and a recall phase spaced 12 hr apart, with participants randomly assigned to two groups: a wake group (who took part in the learning phase at 9 a.m., and recall phase at 9 p.m.), and a sleep group (who took part in the learning phase at 9 p.m. and recall phase at 9 a.m. the following day). Figure 1 illustrates the design. In the wake group, participants were instructed to stay awake during the next 12 hr and refrain from any form of sleep. In the sleep group, participants were instructed to go to bed and sleep. To ensure that participants completed the experiment at set times, the link was only available for 15 min around the target time (9 a.m. or 9 p.m.). Failure to complete the experiment during this window resulted in participants’ data being withdrawn from analysis.

In the learning phase, participants completed the demographic questionnaire, the Mathematics Self-Efficacy and Anxiety Questionnaire (MSEAQ), the SSS, the PSQI, and the total amount slept the night before the experiment before starting the lecture. After completion of the questionnaires, participants were asked to write down everything they knew about the chosen topic with the open-ended question on *p*-values. Any participants who indicated any knowledge of the topic were removed from the analysis. After this, on a separate page, participants could begin the video lecture. Midway through the lecture, an attention check appeared to ensure participants were actively watching, i.e., completing three simple math problems displayed on screen. Participants who failed the attention check were not included in the analysis. After the lecture had finished, participants were asked the same open-ended question on *p*-values. This marked the end of the learning phase.

In the recall phase, all participants completed a second SSS on their sleepiness in the moment, the sleep group reported their sleep, wake, and total sleep times, whereas the wake group confirmed they had not slept in the past 12 hr, with this reported in the “Results” section. Participants were then given 20 min to complete the same open-ended question as from the learning phase, asking them to recall all the information they could about *p-*values. This marked the end of the participant’s involvement in the study. All questionnaires and tasks were completed online on Qualtrics (Qualtrics, Provo, UT). Participant’s answers to the open-ended questions were put through Turnitin (Turnitin, LLC) to detect any plagiarism.

### CJ task

Participants’ answers from each session were arranged into two identical lists collapsed across groups and conditions. These were then randomly shuffled into pairs of answers (one answer from each list). Each answer was, therefore, presented twice in different pairs for a total of 118 pairs per marker to achieve high reliability, in line with the suggestion by Verhavert et al. (2019). Markers were blind to the group and the session of the answers, and each marker was assigned to a unique set of pairings. Across all markers, all answers were presented 20 times. Ten markers (*M* = 30.3, *SD* = 3.4) were recruited through prolific.co to score the written responses on the topic of *p*-values. All markers had a PhD in Psychology, taught research methods at a higher education institution, and taught the concept discussed.

**Figure 2.**
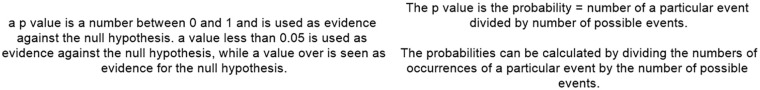
Example of the comparative judgement task. Participants were presented with two answers given by participants and had to judge which was a better explanation of the concept.

In each trial of the CJ task, two written responses were presented side by side on the screen (see Figure 2). Markers had to judge which was the better answer to the question “*Explain what a p-value is and how it is used to someone who hasn’t encountered it before in as much detail as you can.*” If, in the recall phase, a participant did not write anything, their answer was presented as *BLANK*. Markers chose which answer was better using the left or right key. The experiment was built on PsychoPy v3.1.1 (Peirce et al., 2019) and presented via pavlovia.org.

## Results

Thirteen participants were removed from data analysis due to having a PSQI score higher than the cut-off value of 7, meaning 59 participants remained (28 sleep group, 31 wake group). Participants’ average bedtime was 23 hr 15 min, average sleep latency was 29 min, wake time was 7 hr 32 min, and total sleep time was 7 hr 27 min.

Overall PSQI score indicated participants did not significantly differ between sleep (*M* = 4.46, *SD* = 0.99) and wake group (*M* = 3.97, *SD* = 1.19; *t*(57) = 1.72, *p* = .091, *d* = 0.44; see Table 1). MSEAQ scores did not differ between the sleep group (*M* = 78.39, *SD* = 14.76) or the wake group (*M* = 80.65, *SD* = 27.13; *t*(57) = 0.39, *p* = .698, *d* = 0.10). There was no significant difference in SSS score in Session 1 between sleep group (*M* = 1.86, *SD* = 0.71) or wake group (*M* = 1.1, *SD* = 0.62; *t*(57) = 1.42, *p* = .161, *d* = 0.37). There was also no significant difference in SSS score in Session 2 between sleep group (*M* = 1.75, *SD* = 0.52) or wake group (*M* = 1.84, *SD* = 0.45; *t*(57) = 0.70, *p* = .486, *d* = 0.18).

**Table 1.** Means (standard deviation) for sleep measures (PSQI), mathematics anxiety questionnaire (MSEAQ) and comparative judgement score for each session.

	*N*	PSQI score	MSEAQ	Session 1 CJ score	Session 2 CJ score
Sleep group	28 (22 F)	4.46 (0.99)	78.39 (14.76)	0.32 (1.00)	0.47 (1.00)
Wake group	31 (19 F)	3.97 (1.19)	80.65 (27.13)	–0.12 (0.85)	–0.59 (0.83)

PSQI: Pittsburgh Sleep Quality Index; MSEAQ: Mathematics Self-Efficacy and Anxiety Questionnaire; CJ: Comparative Judgement.

We collected 1,180 individual binary CJ choices and submitted these to the Bradley–Terry model (Firth, 2005) to calculate a parameter estimate (hereafter referred to as a CJ score) for each participant’s answer script. The CJ score indicates the probability of each answer being chosen as the better one (with a negative number reflecting a lower probability of being selected as the better answer in a pairing, and a positive number a higher probability of being selected). These scores are then z-transformed to normalise the distribution SSR, a measure of reliability across markers, analogous to Cronbach’s alpha (Bramley, 2007) was high, *r* = .81, suggesting good model fit (Verhavert et al., 2018). Judge misfit values, script misfit values, and dispersion were all within an acceptable range for analysis (see online supplementary information).

**Figure 3.**
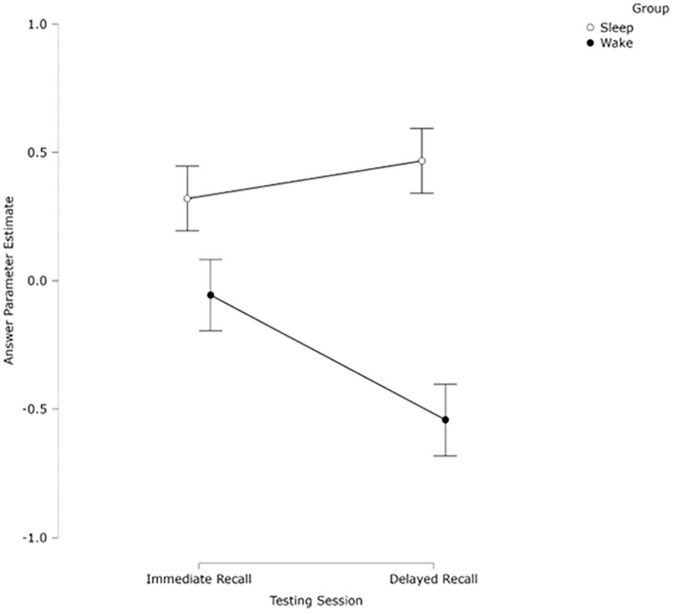
Mean ranked CJ scores of participants’ definition of a *p*-value as judged by experts, split by session and group. Bars indicate ±1 SEM.

A 2 (Group) × 2 (Session) mixed analysis of variance (ANOVA) was conducted on the CJ scores. There was no significant effect of session (*F*(1, 57) = 1.55, *p* = .218, η_p_^2^ = 0.03). There was a significant effect of group (*F*(1, 57) = 13.85, *p* *<* .001, η_p_^2^ = 0.19), with the sleep group (*M* = 0.32, *SD* = 0.99) achieving higher CJ scores than the wake group (*M* = –0.12, *SD* = 0.47). There was also a significant interaction between the session and group (*F*(1, 57) = 5.59, *p* *=* .021, η_p_^2^ = 0.09). Simple effects analyses split by session revealed no significant difference between Session 1 (*M* = 0.32, *SD* = 0.99) or Session 2 (*M* = 0.47, *SD* = 1.00) for the sleep group, *p* = .418, *d* = 0.155, but there was a significant difference for the wake group, *p* = .019, *d* = 0.45, with Session 1 (*M* = –0.12, *SD* = 0.85) scoring significantly higher than Session 2 (*M* = –0.59, *SD* = 0.83). Simple effects analyses split by group revealed no significant difference between the sleep and wake group in Session 1, *p* = .075, *d* = 0.47, suggesting no time-of-day effect for initial learning but it was close to significance, but a significant difference in Session 2, *p* < .001, *d* = 1.15, with the sleep group rated significantly higher than the wake group (see Figure 3).

The results of Experiment 1 suggest there was a significant effect of sleep, with participants in the sleep group being rated as significantly more knowledgeable about *p*-values than those in the wake group.

## Experiment 2: understanding of *t*-tests

### Participants

Sixty-nine participants (56 females, *M* = 23.9, *SD* = 7.8) were recruited from prolific.co (31 participants) or from a first-year undergraduate Psychology statistics module and were unfamiliar with the concept ahead of the experiment. None of the participants from prolific.co had completed a degree in Psychology. All participants were UK-based and had English as their first language. Participants recruited via Prolific.co were paid £8.10 upon completion and undergraduate students received course credits. Undergraduate participants were told that participation in the study was entirely voluntary and did not affect their undergraduate statistics module. The experiment was approved by the University Ethics Board, and written consent was obtained for all participants. Participants did not complete any other experiment within this article. We used the same power analysis and over-recruitment strategy as in Experiment 1. All participants were instructed not to have caffeine and/or alcohol during the period of the experiment, or 12 hr prior, and to refrain from naps during the period of the experiment.

### Stimuli

Stimuli for this experiment mirrored that of Experiment 1. Participants completed the demographic questionnaire, the PSQI (Buysse et al., 1989), the MSEAQ (May, 2009), and the Stanford Sleepiness Scale (Hoddes et al., 1973). The only change to the stimuli was the lecture used.

#### Lecture on *t*-tests

The lecture on *t*-tests was taken from the first-year undergraduate Psychological Research Methods module at the researchers’ University. The module is designed for students with no statistical background to become familiar with common statistics in psychological research. The lecture on *t*-tests was 28 min 34 s long, was presented in English, and addressed what *t*-tests were, how they are used in psychology, and how to interpret them. Midway through the lecture, there was an attention check. Before and after the lecture, participants were asked a single, open-ended question:

Explain what a *t*-test is and how it is used to someone who hasn’t encountered it before in as much detail as you can. If you don’t know what a *t*-test is, please write “I don’t know.”

### Procedure

**Figure 4.**
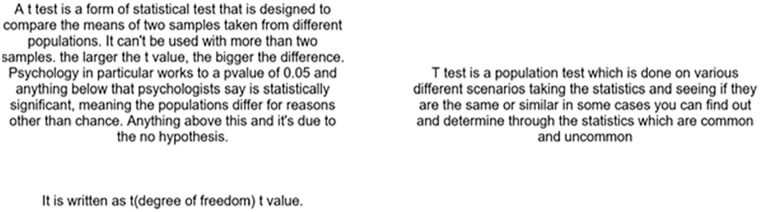
Example of the comparative judgement task and answers provided for Experiment 2. Markers were presented with two answers given by participants and had to judge which was a better explanation of the concept of *t*-test.

The procedure for Experiment 2 was the same as Experiment 1 (see Figure 4).

### CJ task

As per Experiment 1, we pseudo-randomly arranged answers in pairs, and each marker was presented with a different set of 110 pairings. Ten markers (*M* = 29.7, *SD* = 3.5) were recruited through prolific.co to score the written responses on the topic of *t*-tests. All markers had a PhD in Psychology, taught research methods at a higher education institution, and taught the concept discussed. The CJ task was presented as per Experiment 1.

## Results

### Data analysis

Fourteen participants were removed from data analysis due to reporting less than 6 hr sleep or having a PSQI score higher than cut-off (13), or plagiarising answers from an online source (1), meaning 55 participants remained (28 sleep group, 27 wake group). Participants’ average bedtime was 23 hr 05 min, average sleep latency was 22 min, wake time was 7 hr 35 min, and total sleep time was 7 hr 21 min.

Overall PSQI score indicated no significant difference between sleep (*M* = 4.64, *SD* = 1.48) and wake group (*M* = 3.89, *SD* = 1.53; *t*(53) = 1.55, *p* = .125, *d* = 0.42; see Table 2). Overall MSEAQ score did not significantly differ between the sleep group (*M* = 83.36, *SD* = 18.82) or the wake group (*M* = 80.07, *SD* = 22.99; *t*(53) = 0.58, *p* = .565, *d* = 0.16). There was no significant difference in SSS score in Session 1 between sleep group (*M* = 2.11, *SD* = 0.83) or wake group (*M* = 1.89, *SD* = 0.58; *t*(53) = 0.94, *p* = .350, *d* = 0.26). There was also no significant difference in SSS score in Session 2 between sleep group (*M* = 1.96, *SD* = 0.59) or wake group (*M* = 2.08, *SD* = 0.56; *t*(51) = 0.72, *p* = .473, *d* = 0.19).

**Table 2.** Means (standard deviation) for sleep measures (PSQI), mathematics anxiety questionnaire (MSEAQ), and comparative judgement scores for each session.

	N	PSQI score	MSEAQ	Session 1 CJ score	Session 2 CJ score
Sleep group	28 (25 F)	4.64 (1.48)	83.36 (18.82)	–0.07 (1.41)	0.26 (0.96)
Wake group	27 (20 F)	3.89 (1.53)	80.07 (22.99)	0.05 (0.58)	–0.25 (0.82)

PSQI: Pittsburgh Sleep Quality Index; MSEAQ: Mathematics Self-Efficacy and Anxiety Questionnaire; CJ: Comparative Judgement.

Across 1,100 individual CJ choices, we calculated a *z*-transformed parameter estimate for each participant’s script using the Bradley–Terry model (Firth, 2005) as in Experiment 1 and used this CJ score as a measure of conceptual knowledge. Internal consistency among markers measured using SSR was high, *r* = 0.82, suggesting good model fit (Verhavert et al., 2018). Judge misfit values, script misfit values, and dispersion were all within an acceptable range for analysis (see online supplementary information).

**Figure 5.**
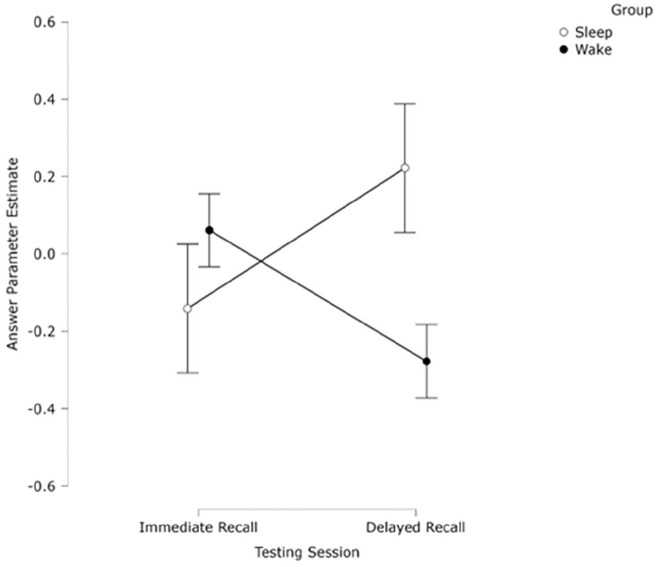
Mean ranked CJ score of participants’ definition of a *t*-test as judged by experts, split by session and group. Bars indicate ±1 SEM.

A 2 (group) × 2 (Session) mixed ANOVA was conducted on the CJ scores. Results showed no significant effect of session (*F*(1, 53) < 1, *p* = .901, η_p_^2^ < 0.01) or of group (*F* <1, *p* *=* .402, η_p_^2^ = 0.01). There was a significant interaction between session and group (*F*(1, 53) = 5.81, *p* *=* .022, η_p_^2^ = 0.10). Simple effects analyses split by session revealed no significant differences between Session 1 (*M* = -0.07, *SD* = 1.41) or Session 2 (*M* = 0.26, *SD* = 0.58) for the sleep group (*p* = .157, *d* = 0.28), but a significant difference between Session 1 (*M* = 0.06, *SD* = 0.59) and Session 2 (*M* = –0.28, *SD* = 0.83) for the wake group (*p* = .035, *d* = 0.43). Simple effects analyses split by group revealed no significant difference between the sleep and wake group in Session 1 (*p* = .686, *d* = 0.11), suggesting no time-of-day effect for initial learning, but there was a significant difference in Session 2 (*p* = .038, *d* = 0.57), with the sleep group scoring significantly higher than the wake group (see Figure 5).

## Experiment 3: understanding of z-scores

### Participants

Sixty-seven participants (60 females, *M* = 22.2, *SD* = 7.1) were recruited through prolific.co (22 participants) or from a first-year undergraduate Psychology statistics module and were unfamiliar with the concept ahead of the experiment. None of the participants from prolific.co had completed a degree in Psychology. All participants were UK-based and had English as their first language. Participants recruited via Prolific.co were paid £8.10 upon completion and undergraduate students received course credits for taking part. Undergraduate participants were told that participation in the study was entirely voluntary and did not affect their undergraduate statistics module. The experiment was approved by the University Ethics Board, and written consent was obtained for all participants. Participants did not complete any other experiment within this article. We used the same power analysis and over-recruitment strategy as in Experiments 1 and 2. All participants were instructed not to have caffeine and/or alcohol during the period of the experiment, or 12 hr prior, and to refrain from naps during the period of the experiment.

### Stimuli

Stimuli for this experiment mirrored that of Experiments 1 and 2. Participants completed the PSQI (Buysse et al., 1989), the MSEAQ (May, 2009), and the Stanford Sleepiness Scale (Hoddes et al., 1973). The only change to the stimuli was the lecture used.

#### Lecture on *z*-scores

The lecture on *z*-scores was taken from the first-year undergraduate Psychological Research Methods module at the researchers University. The module is designed for students with no statistical background to become familiar with common statistics in psychological research. The lecture on *z*-scores was 17 min 29s long, was presented in English, and addressed what *z*-scores were, how they are used in psychology, and how to interpret them. Midway through the lecture, there was an attention check. Before and after the lecture, participants were asked a single, open-ended question:

Explain what a *Z* score is and how it is used to someone who hasn’t encountered it before in as much detail as you can. If you don’t know what a *Z* score is, please write “I don’t know.”

### Procedure

**Figure 6.**
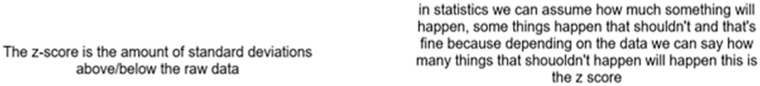
Example of the comparative judgement task and answers provided for Experiment 3. Markers were presented with two answers given by participants and had to judge which was a better explanation of the concept of *z*-score.

The procedure for Experiment 3 was the same as Experiments 1 and 2 (see Figure 6).

### CJ task

Ten markers (*M* = 29.4, *SD* = 3.8) were recruited through prolific.co to score the written responses on the topic of *z*-scores. Each marker completed 106 judgements and pairings of answers were different for each marker. All markers had a PhD in Psychology, taught research methods at a higher education institution, and were familiar with the concept discussed.

## Results

Fourteen participants were removed from data analysis due to plagiarism of their answer from an online source (2) reporting less than 6 hr sleep or having a PSQI score higher than cut-off (12), meaning 53 participants remained (26 sleep group, 27 wake group). Participants’ average bedtime was 23 hr 39 min, average sleep latency was 23 min, wake time was 7 hr 47 min, and total sleep time was 7 hr 21 min.

Overall PSQI score indicated no significant difference between sleep (*M* = 4.31, *SD* = 1.46) and wake group (*M* = 4.67, *SD* = 1.04; *t*(51) = 1.03, *p* = .306, *d* = 0.28; see Table 3). Overall MSEAQ score did not differ between the sleep group (*M* = 84.12, *SD* = 21.45) or the wake group (*M* = 87.00, *SD* = 20.22; *t*(51) = 0.50, *p* = .632, *d* = 0.13). There was no significant difference in SSS score in Session 1 between sleep group (*M* = 2.08, *SD* = 0.89) or wake group (*M* = 1.96, *SD* = 0.52; *t*(51) = 0.57, *p* = .570, *d* = 0.16). There was also no significant difference in SSS score in Session 2 between sleep group (*M* = 1.92, *SD* = 0.63) or wake group (*M* = 2.22, *SD* = 1.12; *t*(51) = 1.19, *p* = .239, *d* = 0.33).

**Table 3.** Means (standard deviation) for sleep measures (PSQI), mathematics anxiety questionnaire (MSEAQ), and comparative judgement score for each session.

	*N*	PSQI score	MSEAQ	Session 1 CJ score	Session 2 CJ score
Sleep group	26 (23 F)	4.31 (1.46)	84.12 (21.45)	0.08 (1.10)	0.25 (0.72)
Wake group	27 (24 F)	4.67 (1.04)	87.00 (20.22)	0.08 (0.87)	–0.40 (1.17)

PSQI: Pittsburgh Sleep Quality Index; MSEAQ: Mathematics Self-Efficacy and Anxiety Questionnaire; CJ: Comparative Judgement.

**Figure 7.**
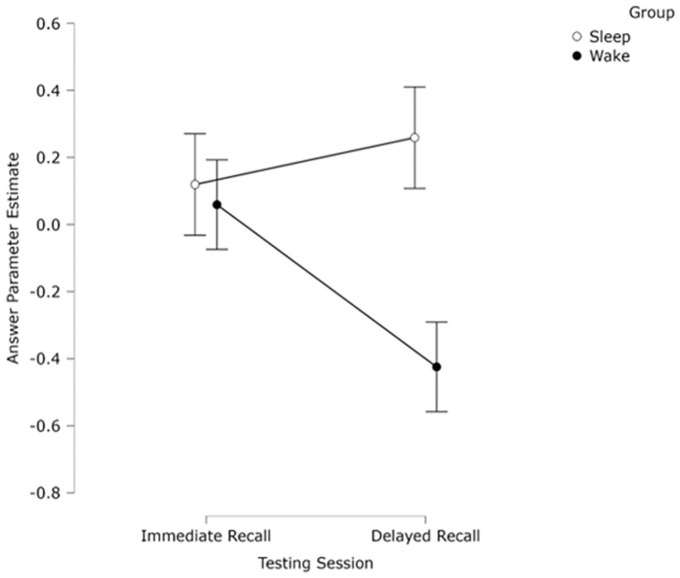
Mean ranked parameter estimate (CJ scores) of participants’ definition of a *z*-score as judged by experts, split by session and group. Bars indicate ±1 SEM.

Across 1,060 individual CJ choices, we calculated a *z*-transformed parameter estimate for each participant’s answer using the Bradley–Terry model (Firth, 2005) as in Experiments 1 and 2 and used this CJ score as a measure of conceptual knowledge (see Figure 7). Internal consistency as measured by the SSR was high, *r* = .85, suggesting good model fit (Verhavert et al., 2018). Judge misfit values, script misfit values, and dispersion were all within an acceptable range for analysis (see online supplementary information).

A 2 (group) × 2 (Session) mixed ANOVA was conducted on the CJ scores. There was no significant effect of session (*F*(1, 51) 1.04 *p* = .312, η_p_^2^ = 0.02), or of group (*F*(1, 51) = 2.12, *p* *=* .152, η_p_^2^ = 0.04). There was a significant interaction between session and group (*F*(1, 51) = 4.49, *p* *=* .039, η_p_^2^ = 0.81). Simple effects analyses split by session revealed no significant difference between Session 1 (*M* = 0.08, *SD* = 1.10) and Session 2 (*M* = 0.25, *SD* = 0.72) for the sleep group (*p* = .478, *d* = 0.14), but a significant difference for the wake group (*p* = .024, *d* = 0.46) Session 1 (*M* = 0.08, *SD* = 0.87), Session 2 (*M* = –0.40, *SD* = 1.17). Simple effects analyses split by group revealed no significant difference between the sleep and wake group in Session 1 (*p* = .99, *d* = 0.01), suggesting no time-of-day effect for initial learning but a significant difference within Session 2 (*p* = .020, *d* = 0.66). Thus, it appears that the significant interaction was driven both by a significant difference within the wake group across sessions and in Session 2 between sleep and wake groups.

## Discussion

This article explored how sleep may affect consolidation of newly learned statistical concepts. After viewing a lecture on a novel concept, participants completed a free-recall task, then after a 12 hr delay containing sleep or wake, participants completed a second free-recall task. All written answers were subject to a CJ task from subject experts to rank the answers based on perceived conceptual understanding, with the resulting scores from Session 1 answers compared to Session 2. Consistent with previous research demonstrating that sleep promotes memory retention (see Diekelmann & Born, 2010; Rasch & Born, 2013 for a review), across three experiments it was observed that conceptual knowledge for a newly learned statistical concept was maintained across a night of sleep, in contrast to a decline in performance across wakefulness. In addition, across all three experiments, there was no significant difference between sleep and wake groups in Session 1, suggesting no initial time-of-day effects were present to influence this. This article is the first to demonstrate the role of sleep in the consolidation of statistical concepts, as well as the first to use CJ as a measure of free-recall memory in a sleep experiment. Below, we discuss this in relation to the existing literature, limitations, and where to move forward.

Within the numerical cognition literature, it is emphasised that although conceptual knowledge and procedural knowledge engage in a bidirectional relationship, with one able to advance the other (Rittle-Johnson & Siegler, 1999), the formation of conceptual knowledge is seen as the foundation to develop procedural knowledge, with the development of conceptual knowledge being a key part of the NCTM’s (2014) plan for mathematical development. The result of the current series of experiments was that across all three experiments, descriptions of the concept given by the sleep group were ranked by subject experts as significantly higher than the wake group after a 12 hr delay period, with no significant difference observed in Session 1. Across all experiments, it was revealed this difference was driven by a significant decline in answer rating between sessions for the wake group, whereas the sleep group maintained similar levels. These results are in line with previous research that has highlighted that sleep can aid in consolidation of newly learned information across a range of domains (see Rasch & Born, 2013 for a review), and more specifically, can aid in the learning of new conceptual knowledge (Graveline & Wamsley, 2017; Hennies et al., 2016) with the sleep-maintaining performance in Session 2, 12 hr later relative to Session 1, rather than enhancing above baseline, whereas the wake group declined. We are unable to say for certain whether this is due to sleep offering passive protection from retroactive interference from new stimuli after learning (Mednick et al., 2011), or whether this aligns more with the active systems consolidation theory that would suggest across both groups participants develop a high-fidelity hippocampal episodic representation of the concept, which they can describe in sufficient detail in immediate recall. It is important to note though that although the experiment is designed as a test of conceptual knowledge, and that CJ tasks have been demonstrated to be able to test this (Bisson et al.,2016, 2020; I. Jones et al., 2019; I. Jones & Davies, 2024), it may be that participants were able to recall information from the lecture without fully understanding the concept at a deeper level, instead recalling the information given to them but with a shallow understanding of the underlying principles. This requires further research. Furthermore, due to participants being tested only 12 hr later and we expect this to still be based upon a hippocampal-based episodic representation, we cannot comment on the possible qualitative transformation to the gist-based representation within the neocortical store emphasised in Active Systems Consolidation theory, something that would be important for the application of the learned statistical concepts to other situations. Although we would expect sleep to demonstrate effects over a longer period (Lutz et al., 2017), we suggest that future research may want to explore this further by adding an additional test component such as requiring participants to interpret a data analysis output. This would be useful to explore whether the understanding of the lecture content can be transferred to a new scenario outside of the initial learning context at a later time point.

### Methodological contributions

This study is, to the authors’ knowledge, the first to utilise a CJ task to measure memory for the concept in relation to sleep. In their meta-analysis, Berres and Erdfelder (2021) identified several factors that can affect the strength of a sleep effect, with repeated testing and type of retrieval, both moderating factors. Previous literature suggests that memory via recall may be more suitable for detecting sleep-related memory effects (Diekelmann & Born, 2010), but the findings are inconsistent. Some studies have reported a beneficial effect of sleep when employing a free-recall task with either story-based (Aly & Moscovitch, 2010) or video-based stimuli (Coutanche et al., 2020), whereas others have observed no effect of sleep on free-recall memory (Cohn-Sheehy et al., 2022; van Rijn et al., 2021). We propose this is, in part, due to how the data were scored. Previous studies typically employ an all-or-nothing scoring measure that can lead to inferences or errors being discarded from analysis losing some nuance of the participants’ answers whereby answers may not fit into a clear category. In contrast, CJ, a methodology that has a strong theoretical background (Thurstone, 1994) and extensive use within educational research (I. Jones & Davies, 2024), does not have this limitation. CJ has been demonstrated to be a reliable and valid measure for marking (I. Jones & Davies, 2024; Pollitt, 2012), requiring little training from knowledgeable markers and offering an efficient way to code responses. Adopting it across the current series of experiments helped mitigate issues with the prior scoring method by relying on expert knowledge of the concept and comparison within participants to make a judgement on the answers. We propose that this method can be adopted more widely within the research field and lead to more studies utilising free-recall tasks for material beyond static stimuli. Previous research into concept learning has primarily used static stimuli and recognition tasks to test participants (Graveline & Wamsley, 2017; Hennies et al., 2016), which has demonstrated a significant effect of sleep. However, these methodologies are not always comparable to real-world learning where information would be often presented in a spoken or written narrative format and then tested via free recall. By adopting a lecture or informative video format alongside CJ future research will be able to explore a wider range of stimuli.

### Limitations

It is important to note that although it was observed that a period containing sleep led to the maintenance of conceptual knowledge, as it was behavioural, it is not possible within this article to say that specific sleep stages led to the observed effects. Previous literature would suggest a role of slow wave sleep (SWS) in consolidation of the concepts (Diekelmann & Born, 2010), with specific sleep architecture, such as sleep spindles and hippocampal sharp wave ripples being linked to memory reactivation (Hennies et al., 2016; Shaw & Monaghan, 2017; Siapas & Wilson, 1998; Sirota et al., 2003; Tamminen et al., 2013). Although within the current series of experiments, we were unable to link specific sleep stages to the maintenance observed, it is something that should be explored in future research.

Similarly, one possibility when comparing these experiments to the existing literature is that although a whole night sleep may benefit retention, it is not clear whether a whole night is necessary. A wide body of nap studies have demonstrated a beneficial effect of sleep on consolidation (see Rasch & Born, 2013); however, within the current experiment, a nap paradigm was impractical. In addition, for real-world learning such as in schools there is not an opportunity to nap after being taught a new mathematical concepts. Instead, one possible avenue for expanding this would be investigating wakeful rest. Research into a short period of non-interference that mimics sleep (often termed awake quiescence, Craig et al., 2018; or wakeful rest, Dewar et al., 2012) has demonstrated a benefit to later retention similar to that of sleep (Craig et al., 2015, 2018; Dewar et al., 2012, 2014; Martini et al., 2020, 2018). In two experiments, Martini et al. (2018) examined the role of wakeful rest in delayed recall, testing retention after 7 days from initial encoding. They reported that an 8 min period of wakeful rest can lead to significant recall 7 days later, but only if participants completed a post-encoding retention task.

## Conclusion

In summary, across three experiments, we report that following a lecture on a novel statistical concept, participants who slept in the following 12 hr maintained a level of conceptual understanding like that of immediately after learning. In contrast, those in the wake group saw a significant decline in their conceptual knowledge over the equivalent period. The results are in line with previous research demonstrating that sleep can be beneficial to newly learned concepts, but this is the first to demonstrate this in relation to statistical concepts and for stimuli learned via a lecture format and free-recall retrieval task using CJ as an assessment. They not only hint at the possibility of a practical benefit from organising study sessions around periods of sleep, but also demonstrate the suitability for CJ in sleep research.

## Supplemental Material

sj-csv-1-qjp-10.1177_17470218251317885 – Supplemental material for Sleep supports the consolidation of newly learned statistical concepts Supplemental material, sj-csv-1-qjp-10.1177_17470218251317885 for Sleep supports the consolidation of newly learned statistical concepts by John J Shaw and Marie-Josee Bisson in Quarterly Journal of Experimental Psychology
